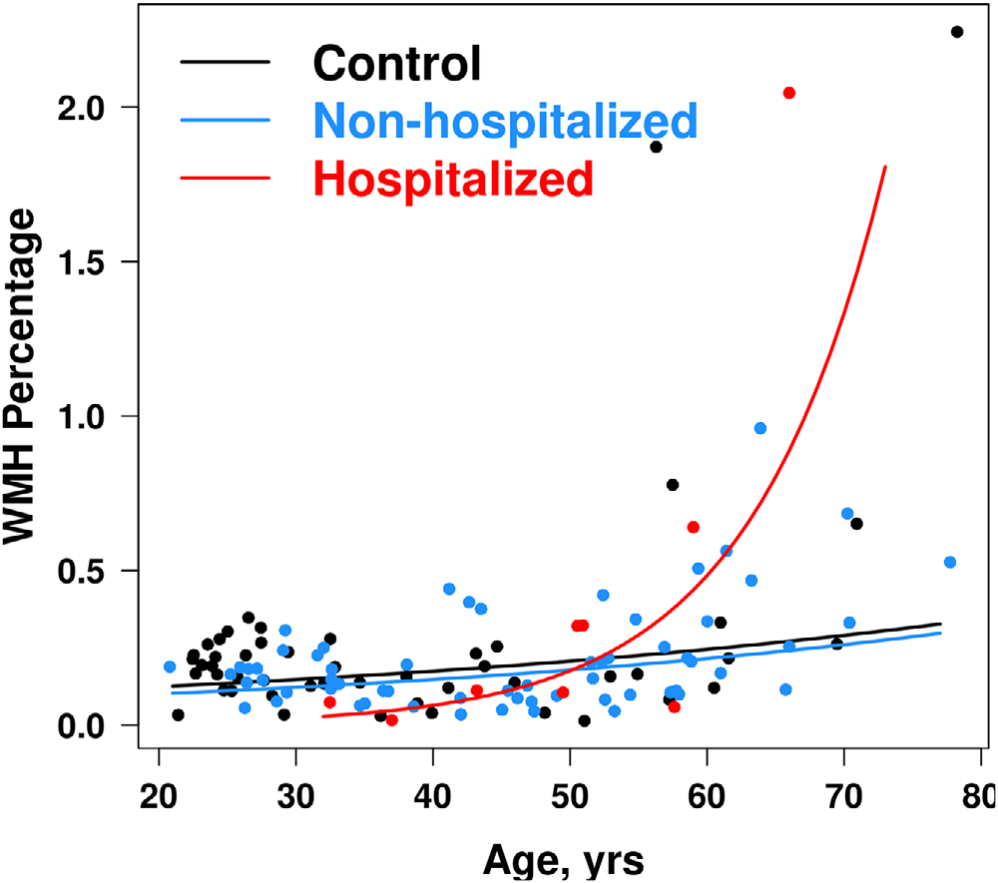# Increase in white matter hyperintensities in long‐COVID depends on age and hospitalization during acute SARS‐CoV‐2

**DOI:** 10.1002/alz.093932

**Published:** 2025-01-09

**Authors:** Merve Atik, June Kendall‐Thomas, Scott A. Przybelski, Orhun H. Kantarci, Burcu Zeydan, Michel Toledano, Matthew L. Senjem, Christopher G. Schwarz, Angela J. Fought, Timothy G. Lesnick, Ana I Silva, James M. Joers, Katie Gundry, Alfredo Lorente, Jeromy Thotland, Dinesh K. Deelchand, Young Woo Park, Xiufeng Li, Georgios Manousakis, Abby Metzler, Christophe Lenglet, Lynn Eberly, Shibani Mukerji, Keenan Christopher Byrne, Meher R Juttukonda, David H Salat, Janet C Sherman, Lauren Pollak, Sevil Yasar, Mehreen Nabi, Sana Rehman, Christof Karmonik, Syed A Gillani, Valerie V Flores, Rachel G Davis, Tetsuo Ashizawa, Eva Maria Ratai, Gülin Öz, Kejal Kantarci, George K Harrold

**Affiliations:** ^1^ Mayo Clinic Graduate School of Biomedical Sciences, Rochester, MN USA; ^2^ Mayo Clinic, Rochester, MN USA; ^3^ Mayo Clinic, Neurology, Rochester, MN USA; ^4^ Mayo Clinic, Radiology, Rochester, MN USA; ^5^ Department of Quantitative Health Sciences, Mayo Clinic, Rochester, MN USA; ^6^ Mayo Clinic, Quantitative Health Sciences, Rochester, MN USA; ^7^ University of Minnesota, Minneapolis, MN USA; ^8^ Massachusetts General Hospital, Boston, MA USA; ^9^ Massachusetts General Hospital, Charlestown, MA USA; ^10^ Johns Hopkins University School of Medicine, Baltimore, MD USA; ^11^ Johns Hopkins University, Baltimore, MD USA; ^12^ Houston Methodist Research Institute, Houston, TX USA; ^13^ The Houston Methodist Research Institute, Houston, TX USA; ^14^ Harvard Medical School/Massachusetts General Hospital, Boston, MA USA; ^15^ Department of Radiology, Mayo Clinic, Rochester, MN USA

## Abstract

**Background:**

Approximately 10% of patients with acute SARS‐CoV‐2 infection present with persistent symptoms recognized as the long‐COVID. Neurological and cognitive symptoms are prevalent in long‐COVID, requiring a deeper understanding of the biological basis of this condition for potential therapeutic interventions. Cerebrovascular complications are observed during acute infection, underscoring the importance of understanding cerebrovascular outcomes. Our objective was to investigate white matter hyperintensities (WMH) as a potential indicator of microvascular disease associated with long‐COVID in the brain

**Method:**

Participants with long‐COVID (n = 71; hospitalized,n = 9,non‐hospitalized,n = 62 during the acute infection) and controls(n = 50) who did not have a reported infection were recruited from the community at five US sites that participated in the COVID‐Brain‐Advanced‐Imaging‐Network (COVID‐BRAIN) project. To be included, participants with long‐COVID had to experience neurological or cognitive sequelae within 6 months after a confirmed infection and continued to show at least one neurological symptom. Clinical evaluation and MRI were performed an average of 20±10 months after the acute infection. WMH volumes were measured from the 3D T2‐weighted FLAIR MRIs using a semi‐automated approach for the WMH segmentation. A linear regression model predicting log transformed WMH volume was used to investigate the association of age, groups (hospitalized long‐COVID, non‐hospitalized long‐COVID, controls), and the interaction of these two predictors along with an adjustment for total intracranial volume. Additionally, predicted regression lines have been plotted on the untransformed WMH scale.

**Result:**

We observed a greater age‐dependent increase in WMH volumes in the hospitalized patients with long‐COVID compared to the control group (group*age interaction, p = 0.005). We did not observe this difference in slopes in the non‐hospitalized patients with long‐COVID compared to the controls. Figure shows the slopes of WMH volume as a percentage of the total intracranial volume by age in the three groups.

**Conclusion:**

Our preliminary observation in this ongoing long‐Covid study suggests that hospitalization during acute COVID is associated with the age‐dependent increase in microvascular injury in the white matter. Older adults with acute infection that require hospitalization are more susceptible to cerebrovascular adverse outcomes than controls or those who do not require hospitalization. Impact of such microvascular changes on cognitive outcomes and persistence of post‐COVID symptoms is yet to be studied.